# (11,13-Dimethyl-1,4,7,10-tetra­azacyclo­trideca-10,13-dienato)copper(II) per­chlorate

**DOI:** 10.1107/S1600536809001664

**Published:** 2009-01-17

**Authors:** Xiao-qiang He

**Affiliations:** a Chemical Technology & Medicine College, Jingchu University of Technology, Jingmen, Hubei 448000, People’s Republic of China

## Abstract

The title complex, [Cu(C_11_H_21_N_4_)]ClO_4_, comprises [Cu^II^(*L*)]^+^ (*L* = 11,13-dimethyl-1,4,7,10-tetra­azacyclo­trideca-10,12-dien­ate) cations and a perchlorate anion. The Cu atom is located on a twofold crystallographic symmetry axis and is coordinated by four N atoms in a slightly distorted square-planar geometry. Inter­molecular N—H⋯O hydrogen bonds are present.

## Related literature

For macrocyclic ligands containing four N atoms in a square-planar coordination geometry, see: Andrews *et al.* (1999 ▶); Kim *et al.* (2004 ▶); Richardson & Sievers (1972 ▶).
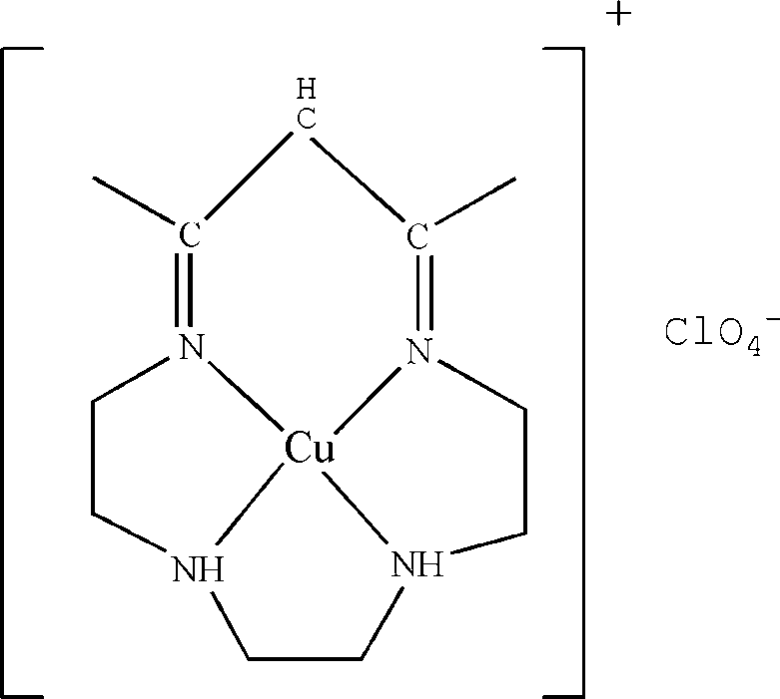


## Experimental

### 

#### Crystal data

[Cu(C_11_H_21_N_4_)]ClO_4_
*M* *_r_* = 372.31
Orthorhombic, 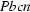

*a* = 12.530 (5) Å
*b* = 14.469 (6) Å
*c* = 8.501 (4) Å
*V* = 1541.2 (11) Å^3^
*Z* = 4
Mo *K*α radiation
μ = 1.61 mm^−1^
*T* = 293 (2) K
0.15 × 0.13 × 0.11 mm

#### Data collection

Bruker APEXII CCD area-detector diffractometer
Absorption correction: multi-scan (*SADABS*; Sheldrick, 2003 ▶) *T*_min_ = 0.794, *T*_max_ = 0.843
6834 measured reflections
1349 independent reflections
950 reflections with *I* > 2σ(*I*)
*R*_int_ = 0.064

#### Refinement

*R*[*F* ^2^ > 2σ(*F* ^2^)] = 0.067
*wR*(*F* ^2^) = 0.216
*S* = 1.05
1349 reflections
98 parameters
H-atom parameters constrained
Δρ_max_ = 1.10 e Å^−3^
Δρ_min_ = −0.59 e Å^−3^

### 

Data collection: *APEX2* (Bruker, 2004 ▶); cell refinement: *SAINT-Plus* (Bruker, 2001 ▶); data reduction: *SAINT-Plus*; program(s) used to solve structure: *SHELXS97* (Sheldrick, 2008 ▶); program(s) used to refine structure: *SHELXL97* (Sheldrick, 2008 ▶); molecular graphics: *XP* (Sheldrick, 1998 ▶); software used to prepare material for publication: *SHELXL97*.

## Supplementary Material

Crystal structure: contains datablocks global, I. DOI: 10.1107/S1600536809001664/hg2468sup1.cif

Structure factors: contains datablocks I. DOI: 10.1107/S1600536809001664/hg2468Isup2.hkl

Additional supplementary materials:  crystallographic information; 3D view; checkCIF report

## Figures and Tables

**Table 1:** Hydrogen-bond geometry (Å, °)

*D*—H⋯*A*	*D*—H	H⋯*A*	*D*⋯*A*	*D*—H⋯*A*
N2—H2⋯O1^ii^	0.91	2.02	2.917 (8)	168

Symmetry codes: (i) 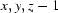; (ii) 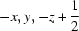.

**Fig. 1.**
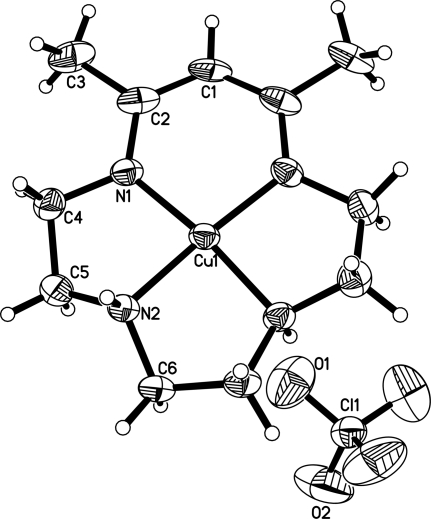
A view of the cation of the title complex with the atom-labelling scheme. Displacement ellipsoids are drawn at the 30% probability level.

## supplementary crystallographic information

### Comment

Macrocyclic ligands containing four N atoms located in a square planar coordination geometry have been intensly studied during the past several decades (Richardson, *et al.*, 1972; Andrews, *et al.*, 1999; Kim, *et al.*, 2004). Here,we report a macrocycle tetraaza copper(II) complex based on the condensation of triethylenetetraamine (trien) with acetylacetone in the presence of copper(II) perchlorate.

The geometry and labelling scheme for the crystal structure of the title complex are depicted in Figure 1. The coordination sphere for the Cu^II^ ion in the title complex, which coordinates to four N atoms from triethylenetetraamine, is square planar with a mean deviation from the plane formed by the Cu atom and four N atoms of 0.0929Å .  The bond lengths of Cu1—N1 and Cu1—N2 are 1.809 (4) and 1.876 (4)Å , respectively, which are slightly shorter than the corresponding distances found in another macrocycle copper complex 13,14-benzo-2,4,9,11-tetramethyl- 1,5,8,12-tetraazacyclotetradeca-1,3,9,11-tetraenato(2-)copper(II) (Kim, *et al.*, 2004).

### Experimental

The title complex has been synthesized by the template method used commonly for synthesize metal complexes of macrocycle ligand. After the mixture, which is formed by acetylacetone, trien and Cu(ClO_4_)_2_.6H_2_O with a molar ratio 1:1:1 in methanol/water(v:v, 1:1), has been stirred for 6 h at 323 K, it was filtered and the filtrate was allowed to partial evaporate in air for one week to produce crystals suitable for X-ray diffraction with a yield of about 45%.

### Refinement

All the H atoms were constrained with C—H distances of 0.93, 0.96, 0.97 Å and N—H distances of 0.91 Å, respectively, and were allowed for as riding atoms with *U*_iso_(H) = 1.2*U*_eq_ (C or N) and 1.5*U*_eq_(C).

### Crystal data

**Table:**

[Cu(C_11_H_21_N_4_)]ClO_4_	*F*(000) = 772
*M**_r_* = 372.31	*D*_x_ = 1.605 Mg m^−^^3^*D*_m_ = 1.605 Mg m^−^^3^*D*_m_ measured by not measured
Orthorhombic, *P**b**c**n*	Mo *K*α radiation, λ = 0.71073 Å
Hall symbol: -P 2n 2ab	Cell parameters from 1838 reflections
*a* = 12.530 (5) Å	θ = 3.2–24.7°
*b* = 14.469 (6) Å	µ = 1.61 mm^−^^1^
*c* = 8.501 (4) Å	*T* = 293 K
*V* = 1541.2 (11) Å^3^	Block, blue
*Z* = 4	0.15 × 0.13 × 0.11 mm

### Data collection

**Table:**

Bruker APEXII CCD area-detector diffractometer	1349 independent reflections
Radiation source: fine-focus sealed tube	950 reflections with *I* > 2σ(*I*)
graphite	*R*_int_ = 0.064
φ and ω scans	θ_max_ = 25.0°, θ_min_ = 2.2°
Absorption correction: multi-scan (*SADABS*; Sheldrick, 2003)	*h* = −14→12
*T*_min_ = 0.794, *T*_max_ = 0.843	*k* = −15→17
6834 measured reflections	*l* = −9→10

### Refinement

**Table:**

Refinement on *F*^2^	Primary atom site location: structure-invariant direct methods
Least-squares matrix: full	Secondary atom site location: difference Fourier map
*R*[*F*^2^ > 2σ(*F*^2^)] = 0.067	Hydrogen site location: inferred from neighbouring sites
*wR*(*F*^2^) = 0.216	H-atom parameters constrained
*S* = 1.05	*w* = 1/[σ^2^(*F*_o_^2^) + (0.144*P*)^2^ + 0.9336*P*] where *P* = (*F*_o_^2^ + 2*F*_c_^2^)/3
1349 reflections	(Δ/σ)_max_ < 0.001
98 parameters	Δρ_max_ = 1.10 e Å^−^^3^
0 restraints	Δρ_min_ = −0.59 e Å^−^^3^

### Special details

**Table:**

Geometry. All e.s.d.'s (except the e.s.d. in the dihedral angle between two l.s. planes) are estimated using the full covariance matrix. The cell e.s.d.'s are taken into account individually in the estimation of e.s.d.'s in distances, angles and torsion angles; correlations between e.s.d.'s in cell parameters are only used when they are defined by crystal symmetry. An approximate (isotropic) treatment of cell e.s.d.'s is used for estimating e.s.d.'s involving l.s. planes.
Refinement. Refinement of *F*^2^ against ALL reflections. The weighted *R*-factor *wR* and goodness of fit *S* are based on *F*^2^, conventional *R*-factors *R* are based on *F*, with *F* set to zero for negative *F*^2^. The threshold expression of *F*^2^ > σ(*F*^2^) is used only for calculating *R*-factors(gt) *etc*. and is not relevant to the choice of reflections for refinement. *R*-factors based on *F*^2^ are statistically about twice as large as those based on *F*, and *R*- factors based on ALL data will be even larger.

### Fractional atomic coordinates and isotropic or equivalent isotropic displacement parameters (Å^2^)

**Table:**

	*x*	*y*	*z*	*U*_iso_*/*U*_eq_	
Cu1	0.0000	0.32069 (5)	0.2500	0.0431 (4)	
Cl1	0.0000	0.14239 (18)	0.7500	0.0660 (7)	
O1	0.0525 (6)	0.2000 (6)	0.6429 (9)	0.139 (3)	
O2	0.0806 (8)	0.0904 (4)	0.8238 (11)	0.160 (3)	
N1	0.0904 (3)	0.4014 (3)	0.1568 (5)	0.0481 (11)	
N2	0.0740 (3)	0.2278 (3)	0.1414 (5)	0.0476 (11)	
H2	0.0427	0.2219	0.0453	0.057*	
C1	0.0000	0.5334 (6)	0.2500	0.060 (2)	
H1	0.0000	0.5976	0.2500	0.072*	
C2	0.0803 (4)	0.4932 (3)	0.1651 (7)	0.0579 (15)	
C3	0.1590 (5)	0.5506 (4)	0.0804 (9)	0.078 (2)	
H3A	0.2298	0.5334	0.1122	0.117*	
H3B	0.1471	0.6146	0.1047	0.117*	
H3C	0.1513	0.5412	−0.0308	0.117*	
C4	0.1727 (4)	0.3589 (4)	0.0637 (7)	0.0576 (14)	
H4A	0.2396	0.3916	0.0780	0.069*	
H4B	0.1537	0.3613	−0.0469	0.069*	
C5	0.1840 (4)	0.2618 (4)	0.1151 (8)	0.0597 (14)	
H5A	0.2253	0.2581	0.2114	0.072*	
H5B	0.2193	0.2254	0.0347	0.072*	
C6	0.0587 (5)	0.1407 (4)	0.2241 (7)	0.0574 (15)	
H6A	0.1056	0.1369	0.3147	0.069*	
H6B	0.0732	0.0888	0.1552	0.069*	

### Atomic displacement parameters (Å^2^)

**Table:**

	*U*^11^	*U*^22^	*U*^33^	*U*^12^	*U*^13^	*U*^23^
Cu1	0.0446 (7)	0.0320 (6)	0.0526 (7)	0.000	−0.0004 (3)	0.000
Cl1	0.0883 (17)	0.0572 (14)	0.0526 (13)	0.000	0.0019 (10)	0.000
O1	0.113 (5)	0.182 (6)	0.122 (6)	−0.012 (5)	−0.020 (4)	0.056 (5)
O2	0.244 (10)	0.106 (4)	0.130 (6)	0.077 (6)	−0.015 (7)	0.003 (5)
N1	0.045 (2)	0.039 (2)	0.060 (3)	−0.0019 (17)	−0.004 (2)	0.0077 (19)
N2	0.055 (3)	0.038 (2)	0.049 (2)	0.0060 (18)	0.0024 (19)	0.0023 (17)
C1	0.069 (5)	0.022 (3)	0.090 (7)	0.000	−0.026 (5)	0.000
C2	0.058 (3)	0.041 (3)	0.075 (4)	−0.011 (3)	−0.031 (3)	0.012 (3)
C3	0.078 (4)	0.051 (3)	0.104 (5)	−0.027 (3)	−0.026 (4)	0.020 (3)
C4	0.048 (3)	0.057 (3)	0.068 (4)	−0.002 (2)	0.006 (3)	0.011 (3)
C5	0.053 (3)	0.059 (3)	0.068 (4)	0.010 (2)	0.002 (3)	0.008 (3)
C6	0.070 (4)	0.034 (3)	0.068 (4)	0.007 (3)	0.002 (3)	0.004 (2)

### Geometric parameters (Å, °)

**Table:**

Cu1—N1^i^	1.809 (4)	C1—C2^i^	1.367 (7)
Cu1—N1	1.809 (4)	C1—H1	0.9300
Cu1—N2^i^	1.876 (4)	C2—C3	1.477 (8)
Cu1—N2	1.876 (4)	C3—H3A	0.9600
Cl1—O1^ii^	1.399 (8)	C3—H3B	0.9600
Cl1—O1	1.399 (8)	C3—H3C	0.9600
Cl1—O2	1.407 (8)	C4—C5	1.479 (8)
Cl1—O2^ii^	1.407 (8)	C4—H4A	0.9700
N1—C2	1.337 (6)	C4—H4B	0.9700
N1—C4	1.438 (7)	C5—H5A	0.9700
N2—C6	1.455 (7)	C5—H5B	0.9700
N2—C5	1.481 (7)	C6—C6^i^	1.535 (13)
N2—H2	0.9100	C6—H6A	0.9700
C1—C2	1.367 (7)	C6—H6B	0.9700
			
N1^i^—Cu1—N1	99.6 (3)	C1—C2—C3	120.7 (5)
N1^i^—Cu1—N2^i^	86.43 (19)	C2—C3—H3A	109.5
N1—Cu1—N2^i^	170.85 (17)	C2—C3—H3B	109.5
N1^i^—Cu1—N2	170.85 (17)	H3A—C3—H3B	109.5
N1—Cu1—N2	86.43 (19)	C2—C3—H3C	109.5
N2^i^—Cu1—N2	88.4 (2)	H3A—C3—H3C	109.5
O1^ii^—Cl1—O1	106.9 (7)	H3B—C3—H3C	109.5
O1^ii^—Cl1—O2	111.4 (5)	N1—C4—C5	108.2 (4)
O1—Cl1—O2	105.8 (5)	N1—C4—H4A	110.1
O1^ii^—Cl1—O2^ii^	105.8 (5)	C5—C4—H4A	110.1
O1—Cl1—O2^ii^	111.4 (5)	N1—C4—H4B	110.1
O2—Cl1—O2^ii^	115.3 (7)	C5—C4—H4B	110.1
C2—N1—C4	121.5 (4)	H4A—C4—H4B	108.4
C2—N1—Cu1	124.0 (4)	C4—C5—N2	105.8 (4)
C4—N1—Cu1	114.5 (3)	C4—C5—H5A	110.6
C6—N2—C5	118.9 (4)	N2—C5—H5A	110.6
C6—N2—Cu1	108.5 (3)	C4—C5—H5B	110.6
C5—N2—Cu1	107.2 (3)	N2—C5—H5B	110.6
C6—N2—H2	107.2	H5A—C5—H5B	108.7
C5—N2—H2	107.2	N2—C6—C6^i^	105.3 (4)
Cu1—N2—H2	107.2	N2—C6—H6A	110.7
C2—C1—C2^i^	129.7 (7)	C6^i^—C6—H6A	110.7
C2—C1—H1	115.1	N2—C6—H6B	110.7
C2^i^—C1—H1	115.1	C6^i^—C6—H6B	110.7
N1—C2—C1	121.3 (6)	H6A—C6—H6B	108.8
N1—C2—C3	118.0 (6)		

Symmetry codes: (i) −*x*, *y*, −*z*+1/2; (ii) −*x*, *y*, −*z*+3/2.

### Hydrogen-bond geometry (Å, °)

**Table:**

*D*—H···*A*	*D*—H	H···*A*	*D*···*A*	*D*—H···*A*
N2—H2···Cl1^iii^	0.91	2.81	3.668 (5)	157
N2—H2···O1^i^	0.91	2.02	2.917 (8)	168

Symmetry codes: (iii) *x*, *y*, *z*−1; (i) −*x*, *y*, −*z*+1/2.
